# Extracorporeal carbon dioxide removal (ECCO_2_R) in post lung transplant patients

**DOI:** 10.1051/ject/2025063

**Published:** 2026-06-19

**Authors:** Mariana N. Zavala Gómez, Elisa Chinchilla Lopez, Ricardo Garza Trevino, Paola Borbolla, Sebastian Bernal Heinzel, Gilberto Lozano Miranda, Allan Méndez, Uriel Chavarria Martinez, Manuel Wong, Adrián Camacho Ortiz, Lilia M. Rizo Topete, Sergio Sánchez Salazar

**Affiliations:** 1 Internal Medicine Residents, Department of Health Sciences, Christus Muguerza Health System, UDEM Monterrey Nuevo León México; 2 Clinical Director, Lung Transplant Program. Christus Muguerza Alta Especialidad Monterrey México; 3 Cardiothoracic and Lung Transplant Surgeon, Lung Transplant Program. Christus Muguerza Alta Especialidad Monterrey México; 4 Infectology Lung Transplant Program, Christus Muguerza Alta Especialidad Monterrey México; 5 Nephrology of the Critically Ill Patient, Internal Medicine, Internal Medicine Professor, Department of Health Sciences, Christus Muguerza Health System Monterrey Nuevo León México; 6 Pulmonary and Critical Care Medicine, Lung Transplant Program, Christus Muguerza Alta Especialidad Monterrey México

**Keywords:** Extracorporeal carbon dioxide removal, Hypercapnia, Post-lung transplant patient, Respiratory failure

## Abstract

Extracorporeal carbon dioxide removal (ECCO_2_R) has emerged as a promising adjunctive therapy to mitigate hypercapnia and reduce invasive mechanical ventilation (IMV) settings. We present the cases of two post-lung transplant patients with severe hypercapnia and respiratory failure who were successfully treated with the ECCO_2_R. In both cases, we used a venovenous ECCO_2_R (V-ECCO_2_R) system, employing the Prismalung™ oxygenator integrated into a Prismaflex™ continuous renal replacement therapy (CRRT) platform. This technique proved efficacy in correcting hypercapnia, improving ventilatory parameters, and facilitating lung-protective strategies in post-lung transplant patients with respiratory failure. This technique represents a valuable adjunct to conventional mechanical ventilation, particularly in cases where hypercapnia poses a risk to graft function and patient stability. Further studies are warranted to establish optimal patient selection and refine treatment protocols for ECCO_2_R implementation in critical care settings.

## Background

Acute respiratory failure is one of the leading causes of admission into intensive care units (ICUs) and represents a significant therapeutic challenge, particularly in patients with acute respiratory distress syndrome (ARDS) or exacerbations of chronic obstructive pulmonary disease (COPD). Traditionally, invasive mechanical ventilation (IMV) has been the cornerstone of respiratory support in these patients. However, prolonged IMV use is associated with adverse effects such as barotrauma, volutrauma, and ventilator-induced diaphragm dysfunction, prompting the search for strategies that minimize ventilator-induced lung injury (VILI) [1].

In this context, extracorporeal carbon dioxide removal (ECCO_2_R) has emerged as a promising adjunctive therapy and an advanced therapeutic technique designed to facilitate the removal of carbon dioxide (CO_2_) from the bloodstream in patients with respiratory failure, thereby enabling the reduction of IMV settings. ECCO_2_R employs similar principles to extracorporeal membrane oxygenation (ECMO) but operates at lower blood flow rates and focuses specifically on CO_2_ removal [2]. This approach has been explored in clinical settings such as moderate ARDS, weaning COPD patients from mechanical ventilation, and ultra-protective lung ventilation strategies [3]. Despite its potential benefits, ECCO_2_R presents significant challenges, including an increased risk of bleeding due to anticoagulation requirements, technical limitations, and the need for precise patient selection to maximize its efficacy [4].

Thus, ECCO_2_R may offer significant advantages in post-lung transplant patients, particularly in scenarios where hypercapnia and conventional mechanical ventilation pose a risk to the transplanted graft. We specifically noted the following: it allows effective removal of carbon dioxide with lower ventilatory settings, thereby minimizing barotrauma, volutrauma, and atelectrauma. By reducing the intensity of mechanical ventilation, ECCO_2_R helps protect the transplanted graft from ventilator-induced lung injury, prevents worsening hypercapnia-related acidosis, and facilitates earlier graft recovery. Additionally, it may serve as a bridge in cases of primary graft dysfunction or delayed graft function, offering time for the transplanted lung to adapt while maintaining adequate gas exchange.

While the routine use of ECCO_2_R in post-lung transplant patients is not yet established [5, 6], its application in selected scenarios has shown promising results. In this context, we present two clinical cases in which ECCO_2_R effectively reduced CO_2_ levels and improved ventilatory parameters, highlighting its potential to support lung-protective ventilation strategies and reduce ventilator-associated complications.

### Case 1

A 42-year-old male with a history of a double lung transplant due to pulmonary fibrosis 10 months ago; treated with prednisone 5 mg qDay, tacrolimus 1 mg BID, mycophenolate mofetil 1 g BID, voriconazol 200 mg qDay, azithromycin 250 mg three days per week, trimethoprim sulfamethoxazole 800/160 mg three times per week, and valganciclovir 900 mg qDay. The patient had a history of bronchial stenosis, and metallic stents were placed in the intermediate bronchus via bronchoscopy, using high-pressure balloon dilation and self-expandable nitinol stents. In addition, a stent was placed in the right upper lobe bronchus. Prior to the decision to place the stent, the patient underwent balloon dilatation sessions with diameters of 11–12–13 mm at 3–4.5–6 atm, respectively, and 13–15–16 mm at 3–4.5–6 atm, respectively, on at least three occasions, followed by bronchial occlusion, which ultimately led to the decision to place stents. He was admitted for dyspnea and desaturation requiring oxygen support with high-flow nasal cannulas. At admission, the laboratory reported: Hemoglobin 12.9 g/dL, Leu 7.88 K/µL, Plt 213,000 K/µL, Glu 115, Cr 0.73 mg/dL, LDH 296 U/L, PT 12.5 s, PTT 35.5 s, RCP 73.10 mg/L. Microbiologic testing was negative for CMV, *Legionella, Pneumocystis jiroveci, Coccidioides immitis, Histoplasma, Mycoplasma pneumoniae, Aspergillus, and Mycobacterium tuberculosis*. A pulmonary CT-angiography reported an area of stenosis at the proximal end of the stent in the right main bronchus and a multilobar pneumonic process; antibiotic therapy was initiated with meropenem and colistin. A bronchoscopy and bronchial dilation were performed. Eventually, the stents had migrated to the intermediate bronchus and the right upper lobe, so they were removed. The patient required three additional bronchial dilations during this hospitalization.

**Figure 1:**
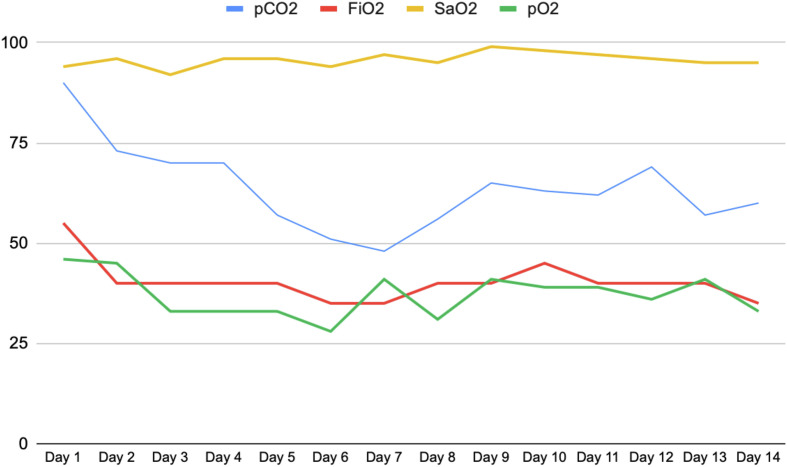
Case 1. Changes in venous CO_2_ and oxygenation parameters during ECCO_2_R.

**Figure 2:**
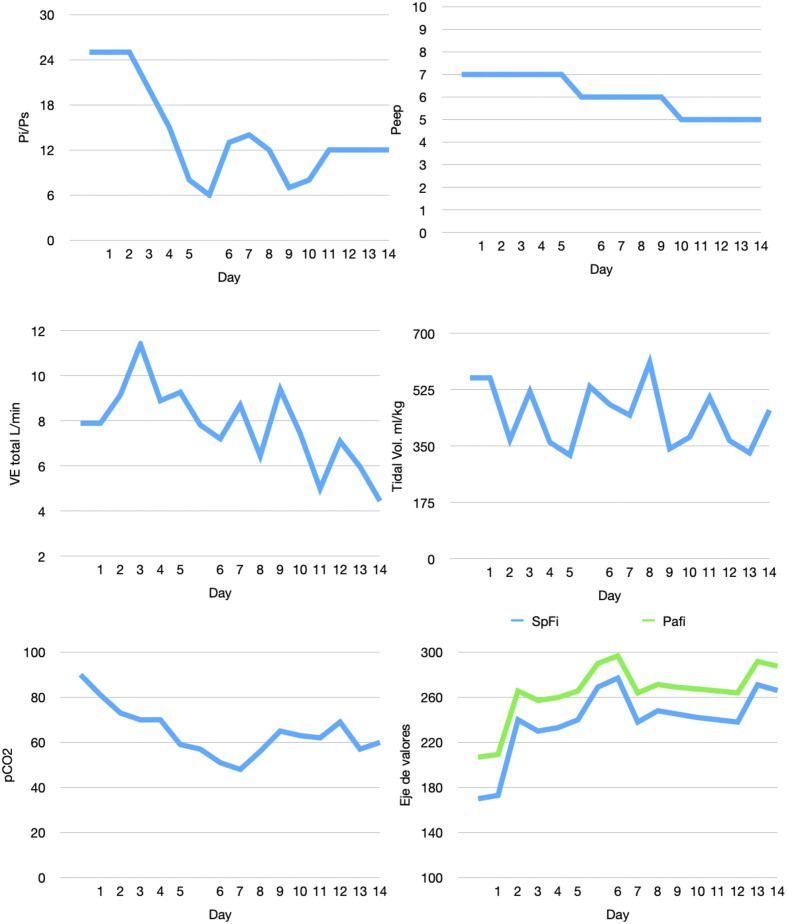
Case 1. Temporal evolution of ventilatory parameters, oxygenation indices, and venous CO_2_ during ECCO_2_R therapy.

His condition subsequently progressed to require IMV due to hypoxemia, hemoptysis, and hypercapnia. A second bronchoscopy showed an adherent clot in the main bronchus that caused obstruction and oozing bleeding in the right upper lobe. He was diagnosed with multidrug-resistant *P. aeruginosa* pneumonia, and cefiderocol was administered for 14 days; concomitantly, immunosuppressive therapy was reduced. A single echocardiogram was performed, which is reported with normal-sized cavities, LV with preserved mobility and thickness. Normal left ventricular systolic function with LVEF 60%, normal diastolic function. Normally right ventricular systolic function. Presence of mild mitral and tricuspid insufficiency. Even with IMV, the patient persisted with hypercapnia, constant *p*CO_2_ levels of 60–94 mmHg with a compensatory metabolic alkalosis. Due to these levels, ECMO was considered as an option for the refractory respiratory failure, but due to costs and potential complications, ECCO_2_R was initiated. ECCO_2_R was performed using a Prismalung™ oxygenator integrated into a Prismaflex™ system. A dual-lumen 13.5 Fr hemodialysis catheter was placed in the right internal jugular vein for vascular access. The circuit included an ST150 filter, with an initial blood flow (Qb) of 200 mL/min. Then, when the ECCO_2_R was added, the average Qb was 400 mL/min and a sweep gas flow of 10 L/min. Systemic anticoagulation with unfractionated heparin was administered at doses ranging from 600 to 900 UI/h to maintain circuit patency, with activated partial thromboplastin time values ranging from 35.1 to 67.6 s. These parameters were selected to optimize CO_2_ removal while ensuring hemodynamic stability and compatibility with the continuous renal replacement therapy (CRRT) circuit. The ventilator settings were pressure control mode (PCV) with an inspiratory pressure (Pi) of 29 cmH_2_O, PEEP of 7 cmH_2_O, respiratory rate (RR) of 20 breaths per minute, inspiratory time (Ti) of 1 s, FiO_2_ of 50%, and a tidal volume of 470 mL. As shown in the graphs, there was a worsening of ventilatory parameters prior to the initiation of ECCO_2_R, with previous settings including Pi 25 cmH_2_O, PEEP 7 cmH_2_O, Ti 0.9 s, FiO_2_ 55%, tidal volume 562 mL, and RR 25 breaths per minute. This support was provided for 14 days, significantly reducing CO_2_ levels, mechanical ventilatory requirements, and offering support and time for treatment and resolution of the pneumonia. (Figures 1 and 2) After the bronchial stenosis was resolved, there was an improvement in ventilatory management, which allowed for the withdrawal of IMV by the 18th day in the ICU.

### Case 2

A 41-year-old female with a history of double lung transplant due to pulmonary arterial hypertension in 2021, was admitted to the general ward for dyspnea and hypoxemia, with increasing oxygen requirements. At admission, the laboratory reported: Hemoglobin 9.7 g/dL, Leu 6.03 K/µL, Plt 77,000 K/µL, Glu 154, Cr 1.45 mg/dL, LDH 307 U/L, RCP 83.40 mg/L, procalcitonin 0.17. Cytomegalovirus, *Pneumocystis jiroveci, Mycoplasma pneumoniae, Aspergillus, and Mycobacterium tuberculosis* were negative. Anti-HLA antibodies Class II were positive and Class I negative. Acute pulmonary embolism was excluded with a CT angiography. A bronchoscopy, bronchial dilation, and transbronchial biopsy were performed on the second day of admission. After the procedure, high-flow nasal oxygen (40 L and FiO_2_ 40%) and methylprednisolone 1 g IV were indicated due to clinical deterioration. The patient continued with worsening dyspnea and tachypnea; invasive mechanical ventilation was indicated on the seventh day of hospital stay. The ventilator settings were pressure control mode (PCV) with a Pi of 23 cmH_2_O, PEEP of 9 cmH_2_O, RR of 28 breaths per minute, Ti of 0.75 s, FiO_2_ of 100%, and a tidal volume of 445 mL. But hypercapnia persisted with a *p*CO_2_ level of 74 mmHg. A transthoracic echocardiogram reported right cavities with significant dilation and concentric remodeling of the left ventricle; LV mobility and thickness were preserved. The systolic function of the left ventricle was normal with LVEF 55%, and diastolic function was not assessed due to sinus tachycardia. Depressed right ventricular systolic function was reported with TAPSE of 14 mm and VTi of 6 cm. Pulmonary artery systolic pressure of 50 mmHg.

**Figure 3:**
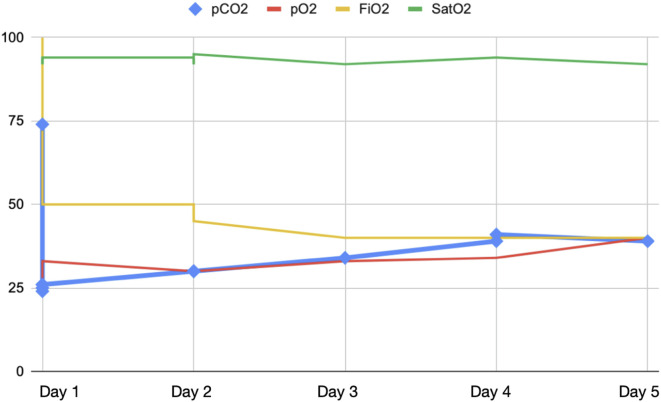
Case 2. Changes in venous CO_2_ and oxygenation parameters during ECCO_2_R.

**Figure 4:**
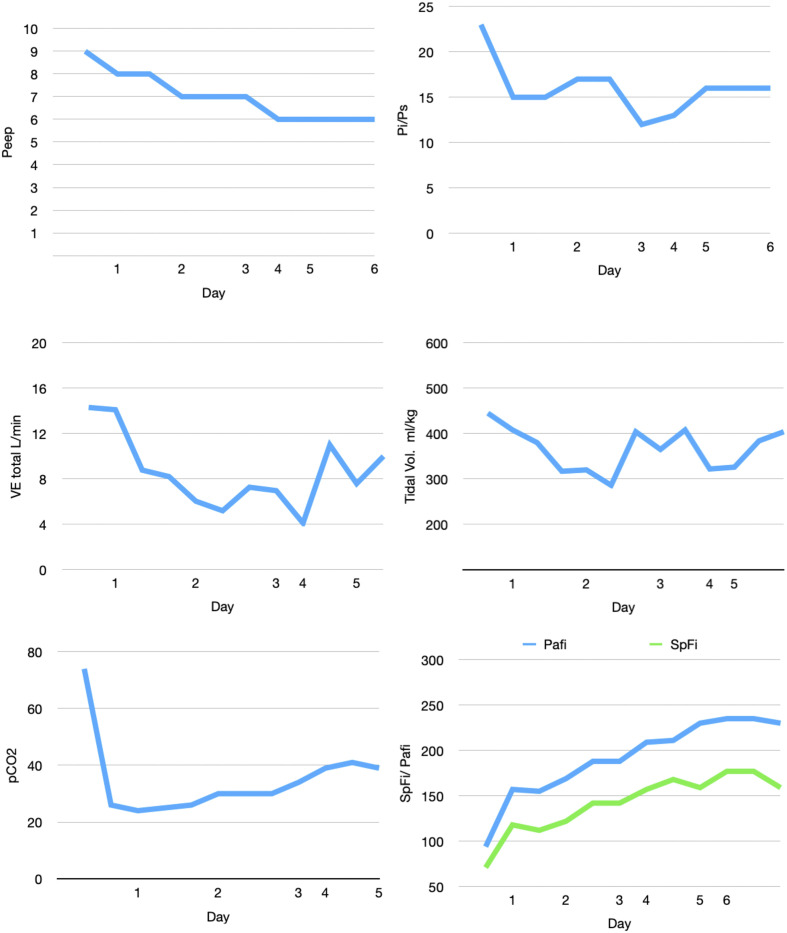
Case 2. Temporal evolution of ventilatory parameters, oxygenation indices, and venous CO_2_ during ECCO_2_R therapy.

Lung biopsy reported histological changes compatible with acute moderate cellular rejection (ISHLT Grade A3). Due to the persistence of respiratory acidosis, ECCO_2_R was initiated using a Prismalung™ oxygenator integrated into a Prismaflex™ system. A dual-lumen 13.5 Fr hemodialysis catheter was placed in the right internal jugular vein for vascular access. The circuit included an ST150 filter, with an average blood flow rate (Qb) of 350-400 mL/min and a sweep gas flow of 8 L/min. Plasma exchange was also indicated as part of treatment for lung transplant mixed rejection, with a volume of 3250 mL, fluid replacement with albumin 5% for 5 days. Prior to the initiation of ECCO_2_R, Pi 20 cmH_2_O, PEEP 8 cmH, FiO_2_ 55%, tidal volume of 317 mL/min. ECCO_2_R support was needed for two days, significantly reducing CO_2_ levels and mechanical ventilatory requirements; a second echocardiogram showed an important improvement of the RV dilation and systolic function with TAPSE of 17 mm and VTi of 16 cm. (Figures 3 and 4) Extubation was successful on the 13th day, and the patient evolved favorably until discharge, going home to continue pulmonary rehabilitation.

**Figure 5:**
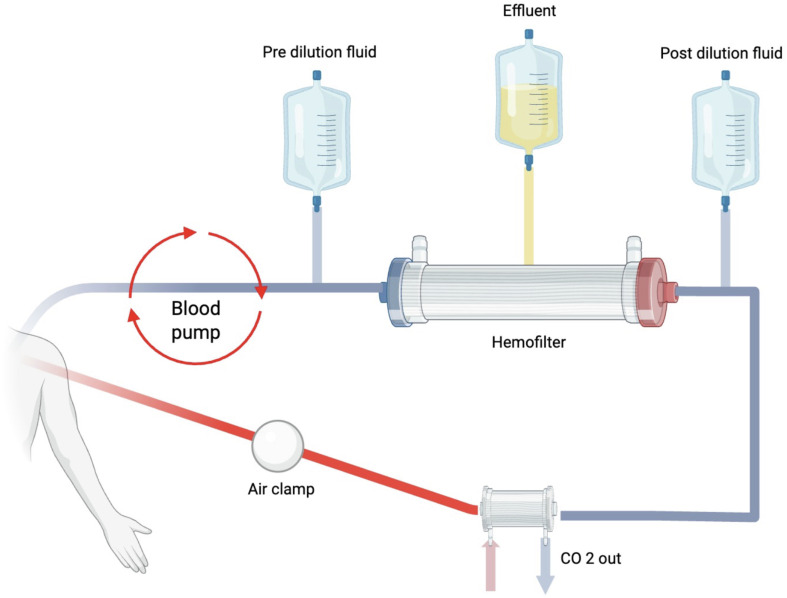
*Hybrid ECCO*_2_*R-CRRT Circuit*. This diagram illustrates a combined extracorporeal circuit in which a continuous renal replacement therapy (CRRT) hemofilter is placed in series with a CO_2_ removal membrane. Blood passes sequentially through the hemofilter for solute and fluid clearance, followed by the ECCO_2_R membrane for gas exchange, before returning to the patient.

**Figure 6:**
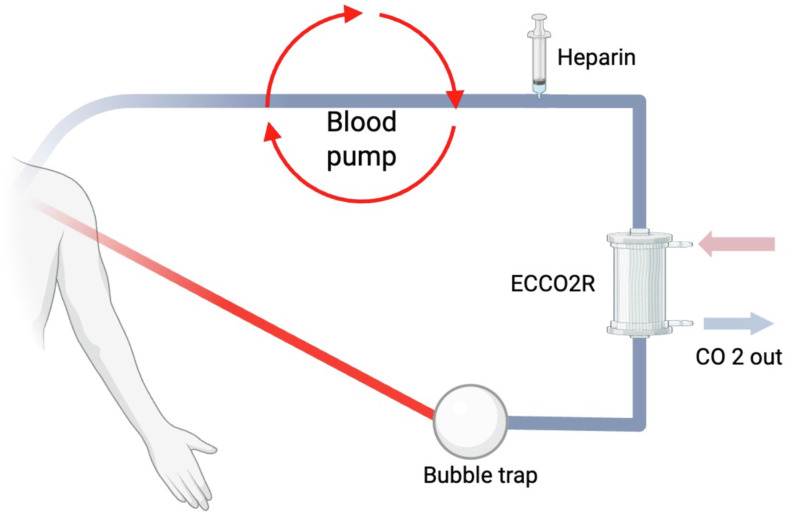
*Standalone ECCO*_2_*R Circuit*. A simplified ECCO_2_R circuit without renal support is depicted. Blood is circulated through a gas-exchange membrane for selective CO_2_ removal, with systemic anticoagulation provided by unfractionated heparin to maintain circuit patency.

## Discussion

Extracorporeal carbon dioxide removal (ECCO_2_R) is an invasive technique developed to manage both hypoxemic and hypercapnic acute respiratory failure. Its primary use was initially conceived for patients with ARDS and those with exacerbated COPD. This CO_2_ removal occurs by using an extracorporeal shunt and gas exchange membrane to remove CO_2_ [7, 8].

In these two cases, both patients had hypercapnia, and high ventilator parameters would have been needed to reach an adequate CO_2_ control. After the start of ECCO_2_R, ventilator support could be reduced, achieving lung-protective parameters while simultaneously lowering CO_2_ levels (as shown in the graphs).

### Technical description of ECCO_2_R

Several ECCO_2_R devices have been developed; they are mainly classified into two types: low-flow, pump-driven venovenous systems (VV-ECCO_2_R) and pumpless arteriovenous systems (AV-ECCO_2_R). In VV-ECCO_2_R, blood is withdrawn from a central vein, passes through a membrane lung for gas exchange, and is returned to the central circulation [9].

The membranes that allow gas exchange are generally composed of hollow fibers made of biocompatible material (poly-4-methyl-1-pentene) with an exchange surface ranging from 0.6 to 2.5 m^2^. They use low blood flows, between 300 and 500 mL/min, which is sufficient for eliminating most of the CO_2_ produced by metabolism. With the development of CO_2_ removal devices, technological advancements have enabled the use of smaller cannulas, typically ranging from 14 to 18 French, depending on the system and settings. Blood is propelled by a nonocclusive roller pump or a centrifugal or diagonal flow magnetic rotary pump, which creates the pressure gradient needed for forward flow through the circuit. It is then directed to a specially designed oxygenator, known as a membrane lung [9, 10]

### Integration of ECCO_2_R into a CRRT System

The integration of extracorporeal carbon dioxide removal (ECCO_2_R) into a continuous renal replacement therapy (CRRT) system offers a unique opportunity to provide both respiratory and renal support in critically ill patients. The main indication for combining CRRT with ECCO_2_R is hypercapnic respiratory acidosis in patients with acute kidney injury (AKI), especially patients with fluid overload, but also patients who meet the criteria and indications according to KDIGO. This combined approach leverages the venovenous hemofiltration circuit to perform both blood purification and CO_2_ removal simultaneously [10].

In a typical ECCO_2_R-CRRT hybrid system, the CO_2_ removal membrane is incorporated in series with the CRRT hemofilter, allowing blood to pass through the dialysis membrane first for solute and fluid clearance, followed by the ECCO_2_R membrane for gas exchange before returning to the patient. There is no proven advantage to placing the oxygenator either before or after the renal filter. The criteria for initiating CRRT remain the same as in AKI without respiratory failure [11]. The key components include [12, 13].

*Blood Pump*: Regulates blood flow (typically between 200 and 500 mL/min) to optimize CO_2_ removal while ensuring adequate renal clearance.

*Membrane Lung* (*Gas Exchange Membrane*): A highly permeable, low-resistance hollow fiber membrane that facilitates CO_2_ diffusion into a sweep gas (typically oxygen or ambient air).

*CRRT Hemofilter*: Removes uremic toxins, excess fluids, and electrolytes according to prescribed settings.

*Sweep Gas System***:** Controls CO_2_ elimination by modulating the gas flow rate, which determines CO_2_ diffusion efficiency.

This dual-function extracorporeal support is particularly beneficial in hemodynamically unstable patients, as it reduces the need for additional vascular access and minimizes excessive extracorporeal blood volume exposure, making it a viable option for patients with cardiorenal and pulmonary-renal syndromes (Figures 5 and 6).

### Multidisciplinary collaboration between pulmonology and nephrology

The successful implementation of ECCO_2_R in CRRT settings requires tight coordination between pulmonologists, nephrologists, and critical care teams to ensure optimal patient selection, monitoring, and therapy adjustments.

### Evidence

Some published trials and consensus have been performed to provide more information and evidence to support this treatment in a wide range of patient groups.

In ARDS, mechanical ventilation may cause VILI. Several lung protective strategies, such as decreasing plateau pressure, driving pressure, power, respiratory rate, or tidal volume, have been suggested to decrease VILI. Further reductions in VT to 3–4 mL/kg may reduce lung stress and strain, alleviate overdistention, and avoid VILI, but very low VT can result in hypercapnia and respiratory acidosis [14].

This hypercapnia and acidosis may cause important side effects. Some of its effects are that it increases cardiac output and heart rate due to sympathetic activation; causes pulmonary hypertension and right ventricular overload; and, on occasion, leads to arrhythmias and reduces cardiac contractility due to acidosis. In the lungs, it causes vasoconstriction in pulmonary arteries, leading to increased pulmonary vascular resistance (PVR) and right ventricular afterload.

The SUPERNOVA study demonstrated that ECCO_2_R facilitated ultra-protective ventilation, mitigating respiratory acidosis in patients with moderate ARDS, and could help mitigate this acidosis by removing CO_2_. They reached a tidal volume of 4 mL/kg PBW in three stages (from 6.0 to 5.0, from 5.0 to 4.5, and from 4.5 to 4 mL/kg PBW). At each stage, PEEP was adjusted to achieve a target P-PLAT of 23–25 cmH_2_O. ECCO_2_R and ultra-protective ventilation were maintained, and data were collected at baseline, 8 h, and 24 h [10].

Another randomized clinical trial, REST, tested a strategy of very low VT, facilitated by low-flow ECCO_2_R, in patients with moderate or severe acute hypoxemic respiratory failure. The trial found no evidence of a benefit in mortality [12]. But a secondary analysis of this trial found that the ventilation ratio has a significant impact on the effectiveness of a strategy that combines very low VT and low-flow ECCO_2_R in reducing mortality. This intervention may lower mortality in patients with a high VR [15].

Another use of ECCO_2_R therapy is that it can help prevent intubation in patients likely to fail non-invasive ventilation (NIV), and facilitate earlier weaning and extubation in those requiring invasive mechanical ventilation [16].

In a European consensus involving 14 experts in critical care and respiratory support with ECCO_2_R or ECMO, the group concluded that the primary goal of ECCO_2_R in ARDS is to provide ultra-protective lung ventilation by controlling CO_2_ levels. Key criteria for starting ECCO_2_R included driving pressure (≥14 cmH_2_O) and plateau pressure (≥25 cmH_2_O). Treatment targets for ARDS included pH (>7.30), respiratory rate (<25 breaths/min), driving pressure (<14 cmH_2_O), and plateau pressure (<25 cmH_2_O). In ae-COPD, ECCO_2_R was recommended when there was no reduction in PaCO_2_ or respiratory rate, with a focus on patient comfort, pH (7.30–7.35), a 10–20% reduction in PaCO_2_, weaning from NIV, and maintaining hemodynamic stability [7].

### Right heart failure and ECCO_2_R

Right heart failure (RHF) is the inability of the right ventricle to pump blood to the lungs, which can lead to increased pulmonary pressures, hypoxemia, and difficulty removing carbon dioxide (CO_2_). In this setting, ECCO_2_R has the potential to improve gas exchange by reducing CO_2_ levels and relieving the burden on both the heart and lungs [10]. The use of ECCO_2_R in patients with RHF is an emerging area of research, and although there are some preliminary studies suggesting benefit, evidence of its effectiveness in this setting is still limited. Some evidence suggests that it improves ventilation and oxygenation. For example, in patients with right heart failure and ARDS, its use has been shown to be effective in reducing CO_2_ levels and preventing the need for IMV [17, 18].

It has also been studied as a support in ARDS in some patients with severe CHF or pulmonary hypertension (PH), by reducing respiratory load and allowing the right ventricle to recover. A 2019 pilot study evaluated the use of ECCO_2_R in patients with heart failure and right ventricular dysfunction and found that this technique could be useful in controlling blood CO_2_ levels, improving oxygenation, and reducing the risk of serious respiratory complications, especially in patients who did not adequately respond to conventional mechanical ventilation [18].

Some studies have found that improved oxygenation and reduced acidosis with ECCO_2_R are associated with improved tricuspid annular plane systolic excursion (TAPSE), because the RV does not have to work as hard to maintain pulmonary circulation. Clinical studies have documented that patients with improved TAPSE may present a reduction in in-hospital mortality and adverse event rates. Right ventricular velocity time integral (VTI) is a marker of right ventricular systolic function. A low VTI indicates a decrease in right ventricular systolic function. Research in patients with RHF and PH or respiratory failure observed that reducing CO_2_ levels and improving oxygenation can relieve the stress on the RV in pumping blood to the lungs, and this may result in an improvement in VTI [18].

In these two patients, TAPSE and VTI were followed by portable ultrasound ecocardioscopy, and an improvement in both heart function markers was noted after the start of ECCO_2_R (Table 1).

**Table 1:** Right Heart Failure improvement during ECCO_2_R by ecocardioscopy.

Day	TAPSE	VTI
1st Case		
1	14	9
2	17	11
3	17	12
2nd Case		
1	12	8
2	14	6
3	17	16
4	18	16
5	22	15

Prospects for the use of ECCO_2_R in patients with CHF are promising, but depend on the development of technology, performance of larger clinical studies, and the identification of ideal patient profiles who would most benefit from this treatment. As the pathophysiology of CHF and its relation to respiratory dysfunction is further understood, specific biomarkers may be identified that help predict who will benefit most from ECCO_2_R [19].

## Conclusion

ECCO_2_R proved to be an effective therapy in two patients by significantly reducing CO_2_ levels and improving ventilatory parameters. This technique facilitated the correction of hypercapnia and pH imbalances, enhancing respiratory stability while minimizing the strain on the lungs. Also, its benefits extend to the right heart, achieving better function. In two cases of lung transplant patients, ECCO_2_R allowed the achievement of protective ventilatory parameters and preserved right ventricular function by improving hypercapnia, while other measures were implemented to address the primary issue. ECCO_2_R offers notable advantages, such as allowing ultra-protective ventilation strategies, reducing the risk of ventilator-induced lung injury, and supporting patients at risk of NIV failure. These findings highlight ECCO_2_R as a valuable adjunct in the management of acute respiratory failure, particularly in cases characterized by severe hypercapnia.

## Data Availability

All relevant data supporting the findings of this study are included within the article. Additional information is available from the corresponding author upon reasonable request.
